# *Ugandaltica* gen. n., a tiny flea beetle from the forest canopy in Central Africa (Coleoptera, Chrysomelidae, Galerucinae, Alticini)

**DOI:** 10.3897/zookeys.746.23637

**Published:** 2018-03-27

**Authors:** Paola D’Alessandro, Maurizio Biondi

**Affiliations:** 1 University of L’Aquila, Department of Life, Health and Environmental Sciences, Section of Environmental Sciences, Via Vetoio, 67100 L’Aquila, Italy

**Keywords:** Afrotropical region, canopy, Coleoptera, Chrysomelidae, ecology, morphology, new genus, new species, taxonomy

## Abstract

In this contribution, *Ugandaltica wagneri* **gen. n.** and **sp. n.**, collected from the canopies in the Budongo Forest, Uganda, is described. Similarities and affinities with other small-sized and convex-shaped flea beetle genera, occurring in the Afrotropical region, are discussed. Micrographs of diagnostic characters, including male and female genitalia, are supplied. Finally, some considerations on the ecology of canopy flea beetles are also reported.

## Introduction

Alticini are a tribe of small to medium sized Coleoptera in the family Chrysomelidae, subfamily Galerucinae, along with the closely related Galerucini (Bouchard et al. 2011). They are named ‘flea beetles’ because of the presence of a metafemoral extensor tendon that enables them to jump (Furth and Suzuki 1998, Nadein and Betz 2016). Alticini are probably the largest and most diverse tribe of Chrysomelidae, comprising about 600 genera and 8000 species (Nadein 2012, Nadein and Beždek 2014, Insektoid.Info 2017). Some genera are widespread in more than one zoogeographical region, i.e. *Altica* Geoffroy, *Aphthona* Chevrolat, *Chaetocnema* Stephens, *Longitarsus* Berthold, etc., while others are strictly endemic to very limited areas (Biondi and D’Alessandro 2017). Flea beetles feed on stems, leaves or roots, and rarely flowers, both in the adult and larval stages. Host plants are known from almost all the vascular plant families, generally with high levels of specialization and close relation with the vegetation types (Jolivet and Verma 2002; Biondi and D’Alessandro 2008, Biondi et al. 2015). There is a higher species richness of flea beetles in the tropics of the southern hemisphere, even though our knowledge about this tribe is still incomplete for many of these areas (Biondi and D’Alessandro 2010a, 2012, Nadein and Beždek 2014). Based on our current knowledge, the whole Afrotropical region, including Madagascar, hosts about 1600 known species, ascribed to 101 different genera (Biondi and D’Alessandro 2010a, 2010b, 2012, 2013a, 2013b, 2015, 2016, 2017, 2018, Döberl 2010, D’Alessandro et al. 2014, 2016, 2017, Biondi et al. 2017, Biondi personal data); and sub-Saharan Africa, in particular, hosts 85 flea beetle genera of which about 65% are endemic (Biondi and D’Alessandro 2010a, 2012, Biondi 2017, Biondi et al. 2017). One of the most interesting, but still poorly and/or methodologically incorrectly investigated habitats is the forest canopy (Wagner 2000, Basset et al. 2003a, 2003b, Furth et al. 2003, Charles and Basset 2005, Davis et al. 2011). Studies on the arthropod composition of the forest canopies in sub-Saharan Africa have revealed a high proportion of Alticini when compared to other beetles (Wagner 2001).

In this contribution the small flea beetle, *Ugandaltica wagneri* gen. n. and sp. n., is described from Budongo Forest, a seasonal rain forest in Western Uganda. The similarities and affinities of this new genus with other small, convexly shaped flea beetle genera are discussed. In addition, some considerations on the ecology of canopy flea beetles are reported.

## Materials and methods

Material examined consisted of dried pinned specimens, collected by fogging trees during the research activities for the Budongo Forest Project (Wagner 2000, 2001, Budongo Conservation Field Station 2017). Specimens were examined, measured, and dissected using a Leica M205C binocular microscope. Photomicrographs were taken using a Leica DFC500 camera and Zerene Stacker software version 1.04. Scanning electron micrographs were taken using a Hitachi TM-1000 scanning electron microscope. Males and females were measured to determine the mean, standard deviation and range of some morphometric measurements for each sex. The terminology follows D’Alessandro et al. (2016 fig. 10E) for the median lobe of the aedeagus, Döberl (1986), Suzuki (1988), and Furth and Suzuki (1994) for the spermatheca, and Furth (1982), Furth and Suzuki (1998), and Nadein and Betz (2016) for the metafemoral extensor tendon. Geographical coordinates for localities are reported in DDM format; information included in square brackets has been added by the authors. Lines on the same label are separated by “/” and labels on the same specimen are separated by “//”. Chorotypes follow Biondi and D’Alessandro (2006).

Abbreviations of depositories:

**BAQ** collection of M. Biondi, Dipartimento di Medicina clinica, Sanità pubblica, Scienze della Vita e dell’Ambiente, Università dell’Aquila, Italy;

**BMNH** The Natural History Museum, formerly British Museum (Natural History), London, Great Britain;

**NMPC** Entomologické oddělení Národního muzea, Praha-Kunratice, Czech Republic.

These internationally recognized codens follow the list on The Insect and Spider Collections of the World Website (Evenhuis 2016). Abbreviations of measurements:

**LA** numerical sequence proportional to length of each antennomere;

**LAED** length of aedeagus;

**LAN** length of antennae;

**LB** total length of body (from apical margin of head to apex of elytra);

**LE** length of elytra;

**LP** medial length of pronotum;

**LSPC** length of spermathecal capsule;

**WE** maximum width of elytra together;

**WP** maximum width of pronotum.

Abbreviations of ecological data, referring to fogged trees and the type of forest, as recorded on the original labels; by courtesy of Thomas Wagner:

**C.a.7** *Cynometra alexandri* (Caesalpiniaceae), primary forest;

**R.a.7** *Rinorea beniensis* (Violaceae), primary forest;

**R.a.22** *Rinorea beniensis*, secondary forest, 8m^2^ collecting sheets;

**R.a.48** *Rinorea beniensis* secondary forest;

**R.a.57** *Rinorea beniensis*, primary forest;

**R.a.78N** *Rinorea beniensis*, secondary forest, night;

**T.r.2, T.r.3, T.r.4, T.r.6, T.r.8** *Trichilia rubescens* (Meliaceae), primary forest.

## Taxonomic part

### Ugandaltica gen. n.

Taxon classificationAnimaliaColeopteraChrysomelidae

http://zoobank.org/3118EF6B-DA2C-4F3D-AB9F-BABED77FCC33

#### Diagnosis.

**Figure 1.**
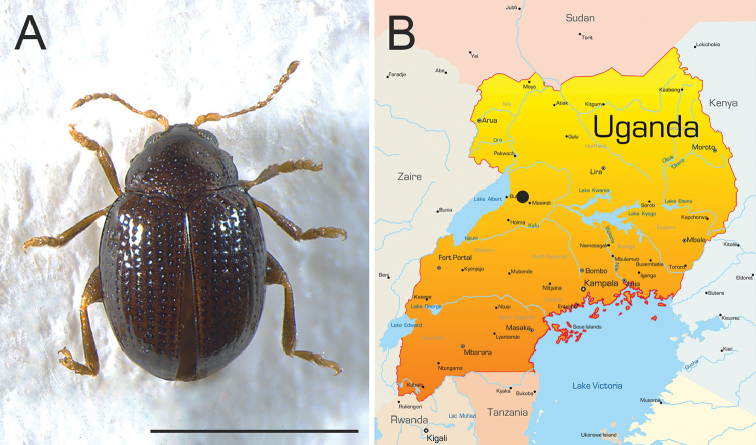
*Ugandaltica wagneri* sp. n. **A** Habitus of male holotype in dorsal view **B** Collecting site (black dot). Scale bar: 1 mm.

The very small size, convex body, and rather short antennae (Fig. 1A) make the new genus similar to the “moss-inhabiting genera”, mainly widespread in the Palaearctic and Oriental Regions (Konstantinov et al. 2013, Damaška and Konstantinov 2016, Ruan et al. 2017). This habitus is, therefore, a clear example of adaptive convergence. The diagnostic characters of the “moss-inhabiting genera” are notably different from those of *Ugandaltica* gen. n., which is also not associated with mosses (see below). Among the Afrotropical flea beetle fauna, *Bezdekaltica* Döberl from Socotra Island (Döberl 2012), *Jacobyana* Maulik from the Oriental and Afrotropical regions (Biondi and D’Alessandro 2011), and *Stegnaspea* Baly from the Republic of South Africa and Tristan da Cunha (D’Alessandro et al. 2012) show a similar general morphology.

The genus *Stegnaspea* is immediately distinguishable from *Ugandaltica* gen. n. by the lack of a scutellum, along with several other characters, concerning among others the head sculpture, shape of the maxillary palpi, shape of the pronotum and pronotal sculpture, elytral surface, male and female genitalia, and the metafemoral extensor tendon (D’Alessandro et al. 2012).

The genus *Jacobyana* shows some similarities with the new genus in its pronotal shape and sculpture, first two antennomeres, and the metafemoral extensor tendon (Biondi and D’Alessandro 2011). However, it is easily distinguishable from this genus by the shape and sculpture of the head, shape of the distal antennomeres, anterior angles of the pronotum, third and fourth visible tarsomeres, and the rather different male and female genitalia.

*Bezdekaltica*, a genus known only from the species *B. socotrana* Döberl, shares along with its general shape (Döberl 2012) some aedeagal characteristics with *Ugandaltica* gen. n., such as the absence of sculpture, the presence of rather big pores on the surface, and the peculiar shape of the phallobasis (Figs 3C, 4C). Notwithstanding these similarities in the aedeagus, considered a likely indicator of real affinity, differences in the female genitalia (Figs 3D, 4D), head and pronotal sculpture, the shape of antennomeres, palpi, prosternum, and the tarsi (Döberl 2012), allow us to consider *Ugandaltica* gen. n. as a different genus. *Bezdekaltica* specifically differs from *Ugandaltica* gen. n. in having: deeply incised frontal grooves which connect in the middle of the frons (Fig. 4A); an anteriorly and posteriorly bordered pronotum, with short longitudinal lateral impressions touching pronotal base (Fig. 4A); sharp maxillary palpi (Fig. 4B); the third tarsomere larger than the second, and slender fourth visible tarsomeres; antennomeres which are clearly more homogenous in shape; and the prosternum wider anteriorly than the intercoxal prothoracic process (Fig. 4B). In addition, *Bezdekaltica* has closed procoxal cavities, this is contrary to the statement reported by Döberl (2012), which also separates it from *Ugandaltica* gen. n. (Fig. 4B).

#### Description.

**Figure 2.**
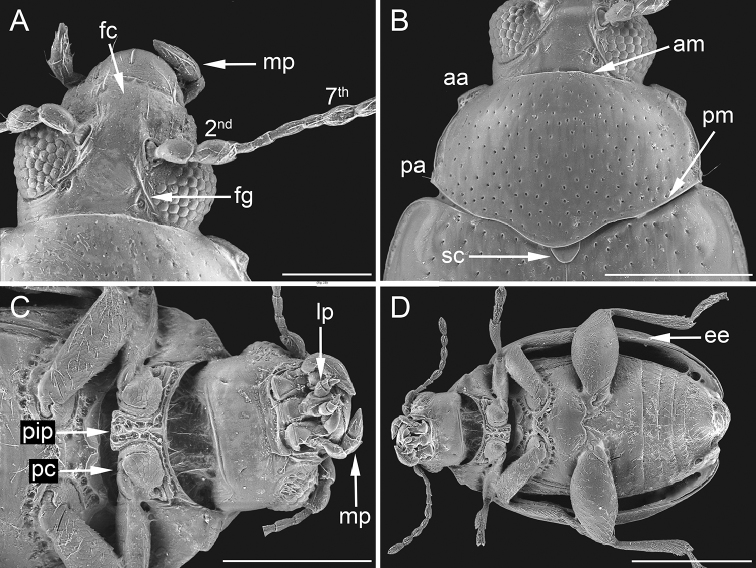
Morphological characters of *Ugandaltica wagneri* sp. n. **A** Head in dorsal view: 2^nd^ = second antennal segment, 7^th^ = seventh antennal segment, fc = frontal carina, fg = frontal groove, mp = maxillary palpus **B** pronotum: aa = anterior angle, am = anterior margin, pa = posterior angle, pm = posterior margin, sc = scutellum **C** Head in ventral view, prosternum and mesosternum: lp = labial palpus, mp = maxillary palpus, pip = prosternal intercoxal process, pc = procoxal cavity **D** Habitus of a female in ventral view: ee = elytral epipleurae. Scale bar: 150 µm (**A**); 300 µm (**B**, **C**); 500 µm (**D**).

**Figure 3.**
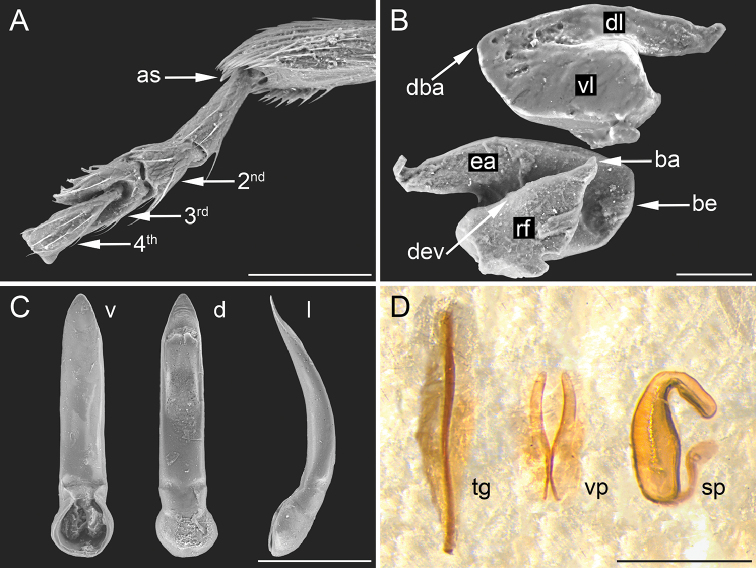
Morphological characters of *Ugandaltica wagneri* sp. n. **A** Distal part of hind tibia, and hind tarsus: 2^nd^ = second tarsomere, 3^rd^ = third tarsomere, 4^th^ = fourth visible tarsomere, as = apical spur of hind tibia **B** Metafemoral extensor tendon: ba = basal angle of ventral lobe, be = basal edge of tendon, dba = dorsal-basal angle of tendon, dl = dorsal lobe, dev = dorsal edge of ventral lobe, ea = extended arm of dorsal lobe, rf = recurve flange, vl = ventral lobe **C** Aedeagus: d = dorsal view, l = lateral view, v = ventral view **D** Female genitalia: sp = spermatheca, tg = tignum, vp = vaginal palpi. Scale bar: 100 µm (**A**); 50 µm (**B**); 200 µm (**C**, **D**)

The new genus is established on the following set of characters. Body roundish, clearly convex, glabrous (Fig. 1A). Frons and vertex smooth; frontal tubercles absent; frontal grooves distinctly impressed, extending from upper ocular margin to antennal socket on each side; genae moderately elongate; maxillary palpi four-segmented, thickset; labial palpi three-segmented (Fig. 2A, C). Antennae slightly shorter than half the body length (Fig. 1A); antennomeres 1-2 distinctly larger than the rest; antennomere 7 distinctly larger than adjacent antennomeres (Fig. 2A). Pronotum sub-trapezoidal, slightly wider posteriorly, distinctly transverse, and distinctly rounded laterally, as wide as elytral base basally; anterior and basal margin not bordered; basal margin distinctly sinuous; lateral margin moderately expanded; anterior angles distinct and prominent, obliquely bevelled; posterior angles with a slightly prominent setigerous pore; punctation clearly impressed (Fig. 2B). Scutellum slightly elongate, roundish apically (Fig. 2B). Elytra indistinctly elongate, entirely covering pygidium, strongly arcuate laterally, jointly rounded apically; lateral margin moderately expanded up to sub-apical part of elytra, slightly visible in dorsal view; punctation arranged in 9 (+ 1 scutellar) regular rows, distinctly impressed but becoming shallower towards the elytral apex; humeral callus distinctly prominent; elytral epipleurae horizontally orientated, slightly visible laterally up to apical fourth of elytra (Figs 1A, 2D). Macropterous metathoracic wings. Hind femora clearly swollen; tibiae straight, not channeled, external margin not dentate; apical spur only present on hind tibiae, simple, and moderately elongate; third tarsomere of all legs about as narrow as second; ungual tarsomere of all legs slightly enlarged; and tarsal claws sub-appendiculate (Figs 2D, 3A). Prosternum clearly narrower anteriorly than intercoxal prothoracic process; and procoxal cavities open (Fig. 2C). Metafemoral extensor tendon with extended arm of dorsal lobe moderately elongate and slightly depressed; basal angle of ventral lobe acute; dorsal margin of ventral lobe straight, distinctly oblique; recurved flange distinctly sclerotized; dorsal-basal angle of the tendon almost right angled; ventral-basal angle of tendon widely obtuse; basal edge of tendon slightly curved (Fig. 3B). The metafemoral extensor tendon of this new genus is similar to those of the *Psylliodes* morpho-group (Furth and Suzuki 1998), but most likely constitutes a new morpho-group, which will include *Jacobyana*. Aedeagal surface without any complex sculpture, but with evident pores; phallobasis widely rounded basally; aedeagus distinctly curved in lateral view (Fig. 3C). Spermatheca of the “alticine type” (Type A of Furth and Suzuki 1994), with distal part distinct from basal part, ductus uncoiled, laterally inserted, and not invaginated in the basal part; neck not distinctly separate from basal part; apical part thicker than neck and distinctly separate (Fig. 3D). Tignum and vaginal palpi as in Fig. 3D.

**Figure 4.**
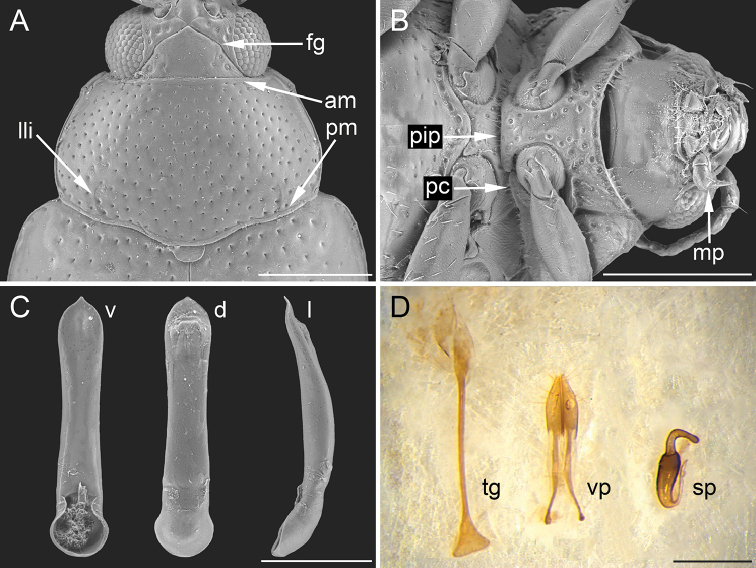
Morphological characters of *Bezdekaltica socotrana* Döberl, paratypes, Yemen, Socotra Island, Dixam plateau, Firmihin, *Dracaena* forest, 12°28.6'N, 54°01.1'E, 490 m, 15–16.xi.2010, J. Bezdĕk leg. (BAQ). **A** pronotum: am = anterior margin, fg = frontal groove, lli = lateral longitudinal impression, pm = posterior margin **B** Head in ventral view, prosternum and mesosternum: mp = maxillary palpus, pc = procoxal cavity, pip = prosternal intercoxal process **C** Aedeagus: d = dorsal view, l = lateral view, v = ventral view **D** Female genitalia: sp = spermatheca, tg = tignum, vp = vaginal palpi. Scale bar: 250 µm (**A**); 300 µm (**B**); 200 µm (**C**); 150 µm (**D**).

#### Etymology.

This new genus is named after Uganda, the country in which it was collected. Female gender.

#### Type species.

*Ugandaltica wagneri* sp. n.

#### Distribution.

Central Africa (Uganda) (Fig. 1B).

### Ugandaltica wagneri sp. n.

Taxon classificationAnimaliaColeopteraChrysomelidae

http://zoobank.org/7A52C5A4-EDAF-48BE-A2B0-EDF0C23C893F

#### Type-specimen.

Holotype male, pinned, with genitalia on the same support. Original label: “Uganda, District Masindi / Budongo Forest n. Sonso / 1°45'N, 31°35'E / 15-25.i.1997 / Th. Wagner leg. [white label] // R.a.78N [white label] // HOLOTYPE / *Ugandaltica wagneri* sp. n. / D’Alessandro and Biondi det. 2018 [red label]” (BAQ).

#### Paratypes.

Uganda, District Masindi / Budongo Forest n. Sonso / 1°45'N, 31°35'E, 19-30.vi.1995 / Th. Wagner leg. // C.a.7, 1 ♂ and 1 ♀ (BAQ); ditto, 1-10.vii.1995 // R.a.7, 1 ♀ (BAQ); ditto, // T.r.2, 1 ♀ (BAQ); ditto, // T.r.3, 1 ♂ (BMNH); ditto, // T.r.4, 1 ♂ (BAQ); ditto, // T.r.6, 1 ♀ (BMNH); ditto, // T.r.8, 1 ♀ (BAQ); ditto, 11-20.vii.1995 // T.r.2, 1 ♀ (BAQ); ditto, 21-30.vii.1995 // R.a.22, 1 ♂ (NMPC); ditto, 5-15.i.1997 // R.a.57, 1 ♀ (NMPC); ditto, 15-25.i.1997 // R.a.48, 2 ♂ and 1 ♀ (BAQ).

#### Type locality.

Uganda, District Masindi, Budongo Forest n. Sonso, 1°45'N, 31°35'E, secondary forest, night, on fogged *Rinorea beniensis*, 15-25.i.1997, Th. Wagner leg.

#### Description of the holotype.

Body small-sized, roundish, distinctly convex (Fig. 1A); LB = 1.20 mm. Maximum pronotal width in basal third (WP = 0.52 mm); maximum elytral width in middle (WE = 0.74 mm). Dorsal integument brownish, shiny, with slightly paler elytral suture. Frons and vertex smooth (Fig. 2A); frontal tubercles absent; frontal grooves distinctly impressed, extending from upper ocular margin to antennal socket on either side; inter-antennal space slightly wider than length of first antennomere; frontal carina wide, flat apically, not delimited laterally, and poorly delimited posteriorly; eye sub-elliptical, rather wide; antennae slightly shorter than half body length (Fig. 1A) (LAN = 0.58 mm; LAN/LB = 0.48); antennomeres (Fig. 2A) 1–2 distinctly wider than the rest, the first sub-conical and the second sub-cylindrical; 7 distinctly wider than the adjacent antennomeres; 1–3 pale in colour, 8–11 brownish; LA: 100:114:43:71:71:71:93:86:100:114:135. Pronotum (Fig. 2B) sub-trapezoidal, slightly wider posteriorly, distinctly transverse (LP = 0.34 mm; WP/LP = 1.51), distinctly rounded laterally, and as wide as elytra basally; not margined anteriorly and basally; basal margin distinctly sinuous; lateral margin moderately expanded; anterior angles distinctly prominent, obliquely bevelled; posterior angles with a slightly prominent setigerous pore; punctation rather dense and uniform; most punctures slightly elongate and deeply impressed on sub-smooth surface. Scutellum (Fig. 2B) slightly elongate, roundish apically, with sub-smooth surface. Elytra (Figs 1A, 2D) slightly elongate (LE = 0.99 mm; WE/LE = 0.75), entirely covering pygidium, strongly arcuate laterally, jointly rounded apically; lateral margin moderately expanded up to sub-apical part of elytra, slightly visible in dorsal view; punctation arranged in 9 (+ 1 scutellar) regular rows; punctures larger than on pronotum, and distinctly impressed on most of the surface (shallower towards elytral apex); interstriae slightly raised, humeral callus distinct and prominent. Macropterous metathoracic wings. Legs with femora dark brown and tibiae brownish; coxae, apex of femora, base of tibiae, and tarsi yellowish. First tarsomere of anterior and middle tarsi distinctly dilated. Body dark brown ventrally; last visible abdominal sternite without special preapical impressions. Aedeagus (Fig. 3C) (LAED = 0.49 mm; LE/LAED = 2.03) tapering slightly towards the apex in ventral view; sub-triangular apically, acute, without a median tooth; surface smooth, with rather large pores, more numerous laterally; phallobasis widely rounded basally; aedeagus distinctly and evenly curved in lateral view, the apex slightly dorsally oriented; dorsal ligula about half the length of median lobe, wide, formed by an apically acute, triangular, median lobe, and two lateral lobes.

#### Variations.

Paratypes are very similar in size, shape, sculpture and colour to the holotype. Female without the dilated first tarsomere in the anterior and middle tarsi. Spermatheca (Fig. 3D) with sub-cylindrical basal part, narrower towards distal part; neck not distinctly separated from basal part; apical part thicker than neck and distinctly separated from it; ductus thin, as long as half the length of basal part, uncoiled, and laterally inserted.

Male (n = 6; mean and standard deviation; range): LE = 1.01 ± 0.10 mm (0.93 ≤ LE ≤ 1.20 mm); WE = 0.76 ± 0.07 mm (0.71 ≤ WE ≤ 0.89 mm); LP = 0.35 ± 0.02 mm (0.34 ≤ LP ≤ 0.39 mm); WP = 0.53 ± 0.04 mm (0.50 ≤ WP ≤ 0.61 mm); LAN = 0.60 ± 0.03 mm (0.58 ≤ LAN ≤ 0.66 mm); LAED = 0.49 ± 0.04 mm (0.46 ≤ LAED ≤ 0.58 mm); LB = 1.27 ± 0.03 mm (1.18 ≤ LB ≤ 1.53 mm); LE/LP = 2.88 ± 0.12 (2.74 ≤ LE/LP ≤ 3.10); WE/WP = 1.43 ± 0.02 (1.40 ≤ WE/WP ≤ 1.46); WP/LP = 1.51 ± 0.04 (1.48 ≤ WP/LP ≤ 1.58); WE/LE = 0.75 ± 0.01 (0.74 ≤ WE/LE ≤ 0.77); LAN/LB = 0.47 ± 0.02 (0.43 ≤ LAN/LB ≤ 0.50); LE/LAED = 2.05 ± 0.08 (1.90 ≤ LE/ LAED ≤ 2.13). Female (n = 6; mean and standard deviation; range): LE = 1.07 ± 0.04 mm (1.00 ≤ LE ≤ 1.13 mm); WE = 0.82 ± 0.02 mm (0.79 ≤ WE ≤ 0.85 mm); LP = 0.36 ± 0.02 mm (0.34 ≤ LP ≤ 0.39 mm); WP = 0.56 ± 0.01 mm (0.55 ≤ WP ≤ 0.58 mm); LAN = 0.59 ± 0.01 mm (0.58 ≤ LAN ≤ 0.61 mm); LSPC = 0.20 ± 0.01 mm (0.19 ≤ LSPC ≤ 0.21 mm); LB = 1.35 ± 0.04 mm (1.29 ≤ LB ≤ 1.40 mm); LE/LP = 2.97 ± 0.10 (2.83 ≤ LE/LP ≤ 3.13); WE/WP = 1.46 ± 0.02 (1.43 ≤ WE/WP ≤ 1.48); WP/LP = 1.55 ± 0.06 (1.48 ≤ WP/LP ≤ 1.61); WE/LE = 0.77 ± 0.02 (0.73 ≤ WE/LE ≤ 0.79); LAN/LB = 0.44 ± 0.02 (0.42 ≤ LAN/LB ≤ 0.46); LE/LSPC = 5.40 ± 0.28 (4.85 ≤ LE/LSPC ≤ 5.67).

#### Etymology.

The specific epithet is a Latinized noun in the genitive case referring to its collector Thomas Wagner (University of Koblenz-Landau, Germany), renowned specialist of Afrotropical Galerucini.

#### Distribution.

Central Africa (Uganda). Considering both the habitat types and the most common species distributions associated with each chorotype (Biondi and D’Alessandro 2006), *Ugandaltica wagneri* sp. n. possibly falls inside the Northern-Eastern Afrotropical chorotype (NEA).

#### Ecology.

All the specimens were collected in primary and secondary forest, at 1200 m a.s.l., by fogging the following trees: *Trichilia rubescens* (Meliaceae), *Rinorea beniensis* (Violaceae), and *Cynometra alexandri* (Caesalpiniaceae). The species was present during both the wet season, June and July 1995, and dry season, January 1997 (Wagner 1999, 2000, 2001).

## Discussion

The general similarities of the new taxon, here described, with those of the “moss-inhabiting genera” seem incidental, and is not due to similar habitat occupancy. In addition, being macropterous is indicative that *Ugandaltica* gen. n. can move easily, a characteristic not found in moss-inhabiting flea beetles (Konstantinov et al. 2013, Damaška and Konstantinov 2016, Ruan et al. 2017). *Ugandaltica wagneri* sp. n. was collected by fogging trees in primary and secondary tropical forest formations (Wagner 1999, 2000, 2001). Wagner (1999, 2001) found that the flea beetle abundance significantly increased in both the primary and the secondary forest during the dry season. The canopies of trees with dense foliage are often the most humid habitats, and small, soft-bodied insects in particular presumably accumulate along a gradient of humidity. Moreover, there is evidence that the abundance of phytophagous insects peaked during leaf flush periods, which happen during the late dry season at the end of January. This is because of their preference for young leaves, and because herbaceous food plants are often no longer available (Wagner 2001). However, Alticini seemed not to feed on trees, as gut dissection of the most abundant species revealed, but rather fed on plants in the surrounding habitat (Wagner 1999). It is interesting that *Ugandaltica wagneri* sp. n. was one of the few flea beetle species also present in the canopy during the wet season. Because of the poor conservation status, and the very small size of the specimens, we preferred not to dissect their gut to investigate whether they were feeding on tree foliage. If they used the canopy as refugium habitat only, then they would only exploit their real host plant during a limited period of the year.

## Conclusions

Most studies on the arthropod composition of the canopy have dealt with several different taxa, which is why a morphospecies approach has often been chosen. This implies that the collected specimens often required further taxonomic investigation by a specialist for their determination. In this paper a new genus and species from a tropical forest in Western Uganda are described, providing a contribution to the knowledge of the flea beetle fauna from canopies of Afrotropical forests. Alticini seem to be one of the more representative taxa of the canopy of tropical forests (Basset and Samuelson 1996, Wagner 1999, 2001, Furth 2003, Charles and Basset 2005). However, because of the dynamics of the canopy faunal composition, the correct interpretation of their presence needs more insight on the ecology and biology of the species found there. For this reason, it will be fundamental to understand how they are distributed in the forest habitat as a whole, and not only in canopy habitats (Basset et al. 2003a). In this regard it must be said that further research in forest habitats might reveal that *Ugandaltica wagneri* sp. n. is more closely associated with a specific forest layer, considering the small number of known specimens and the confined habitat from which they were collected.

## Supplementary Material

XML Treatment for Ugandaltica

XML Treatment for Ugandaltica wagneri

## References

[B1] Basset Y Samuelson GA (1996) Ecological characteristics of an arboreal community of Chrysomelidae in Papua New Guinea. In: Jolivet PHA Cox ML (Eds) Chrysomelidae biology Vol. 2 Ecological studies. SPB Academic, Amsterdam, 243–262.

[B2] Basset Y Hammond PM Barrios H Holloway JD Miller SE (2003a) Vertical stratification of arthropod assemblages. In: Basset Y Novotny V Miller SE Kitching RL (Eds) Arthropods of Tropical Forests: Spatio-Temporal Dynamics and Resource Use in the Canopy. Cambridge University Press, Cambridge, 17–27.

[B3] Basset Y Novotny V Miller SE Kitching RL (2003b) Canopy entomology, an expanding field of natural science. In: Basset Y Novotny V Miller SE Kitching RL (Eds) Arthropods of Tropical Forests: Spatio-Temporal Dynamics and Resource Use in the Canopy. Cambridge University Press, Cambridge, 4–6.

[B4] Budongo Conservation Field Station (2017) History of Budongo. http://www.budongo.org/about/the-history-of-budongo/

[B5] Biondi M (2017) *Hesperoides*, a new “hairy” flea beetle genus from southern Africa (Coleoptera: Chrysomelidae, Galerucinae, Alticini). Fragmenta entomologica 49(2): 151–158. http://www.fragmentaentomol.org/index.php/fragmenta/article/view/257/248, https://doi.org/10.4081/fe.2017.257

[B6] Biondi M D’Alessandro P (2006) Biogeographical analysis of the flea beetle genus *Chaetocnema* in the Afrotropical Region: distribution patterns and areas of endemism. Journal of Biogeography 33: 720–730. https://doi.org/10.1111/j.1365-2699.2006.01446.x

[B7] Biondi M D’Alessandro P (2008) Taxonomical revision of the *Longitarsus capensis* species-group: an example of Mediterranean-southern African disjunct distributions (Coleoptera: Chrysomelidae). European Journal of Entomology 115: 719–736. https://doi.org/10.14411/eje.2008.099

[B8] Biondi M D’Alessandro P (2010a) Genus-group names of Afrotropical flea beetles (Coleoptera: Chrysomelidae: Alticinae): Annotated catalogue and biogeographical notes. European Journal of Entomology 107: 401–424. https://doi.org/10.14411/eje.2010.049

[B9] Biondi M D’Alessandro P (2010b) Revision of the Afrotropical flea beetle genus *Serraphula* Jacoby and description of *Bechynella*, a new genus from Western and Central Africa (Coleoptera: Chrysomelidae: Alticinae). Zootaxa 2444: 1–44.

[B10] Biondi M D’Alessandro P (2011) *Jacobyana* Maulik, an Oriental flea beetle genus new for the Afrotropical Region with description of three new species from Central and Southern Africa (Coleoptera, Chrysomelidae, Alticinae). ZooKeys 86: 47–59. https://doi.org/10.3897/zookeys.86.804 10.3897/zookeys.86.804 PMC3082989 21594092

[B11] Biondi M D’Alessandro P (2012) Afrotropical flea beetle genera: a key to their identification, updated catalogue and biogeographical analysis (Coleoptera, Chrysomelidae, Galerucinae, Alticini). Zookeys 253: 1–158. https://doi.org/10.3897/zookeys.253.3414 10.3897/zookeys.253.3414 PMC3560840 23378812

[B12] Biondi M D’Alessandro P (2013a) The genus *Chabria* Jacoby: first records in the Afrotropical region with description of three new species and annotated worldwide species catalogue (Coleoptera, Chrysomelidae, Galerucinae, Alticini). Zoologischer Anzeiger 252(1): 88–100. https://doi.org/10.1016/j.jcz.2012.03.005

[B13] Biondi M D’Alessandro P (2013b) *Ntaolaltica* and *Pseudophygasia*, two new flea beetle genera from Madagascar (Coleoptera: Chrysomelidae: Galerucinae: Alticini). Insect Systematics & Evolution 44: 93–106. https://doi.org/10.1163/1876312X-04401004

[B14] Biondi M D’Alessandro P (2015) Revision of the Afrotropical genus *Notomela* Jacoby, 1899 with description of *N. joliveti* sp. n. from Principe Island (Coleoptera, Chrysomelidae, Galerucinae, Alticini). In: Jolivet P Santiago-Blay J Schmitt M (Eds) Research on Chrysomelidae 5. ZooKeys 547: 63–74. https://doi.org/10.3897/zookeys.547.9375 10.3897/zookeys.547.9375 PMC4714333 26798314

[B15] Biondi M D’Alessandro P (2016) Revision of *Diphaulacosoma* Jacoby, an endemic flea beetle genus from Madagascar, with description of three new species (Coleoptera: Chrysomelidae, Galerucinae, Alticini). Fragmenta entomologica 48(2): 143–151. https://doi.org/10.4081/fe.2016.181

[B16] Biondi M D’Alessandro P (2017) *Guilielmia* Weise, a little known Afrotropical flea beetle genus: systematic affinities and description of a second new species from Central Africa (Coleoptera, Chrysomelidae, Galerucinae, Alticini). Zootaxa 4323(4): 572–578. https://doi.org/10.11646/zootaxa.4323.4.9

[B17] Biondi M D’Alessandro P (2018) Taxonomic revision of the genus *Angulaphthona* Bechyné (Coleoptera, Chrysomelidae, Galerucinae). European Journal of Entomology 115: 30–44. https://doi.org/10.14411/eje.2018.005

[B18] Biondi M Urbani F D’Alessandro P (2015) Relationships between the geographic distribution of phytophagous insects and different types of vegetation: a case study of the flea beetle genus *Chaetocnema* (Coleoptera: Chrysomelidae) in the Afrotropical region. European Journal of Entomology 112(2): 311–327. https://doi.org/10.14411/eje.2015.040

[B19] Biondi M Frasca R Grobbelaar E D’Alessandro P (2017) Supraspecific taxonomy of the flea beetle genus *Blepharida* Chevrolat, 1836 (Coleoptera: Chrysomelidae) in the Afrotropical Region and description of Afroblepharida subgen. n. Insect Systematics & Evolution 48: 97–155. https://doi.org/10.1163/1876312X-48022152

[B20] Bouchard P Bousquet Y Davies AE Alonso-Zarazaga MA Lawrence JF Lyal CHC Newton AF Reid CAM Schmitt M Slipinski SA Smith ABT (2011) Family-group names in Coleoptera (Insecta). ZooKeys 88: 1–972. https://doi.org/10.3897/zookeys.88.807 10.3897/zookeys.88.807 PMC3088472 21594053

[B21] Charles E Basset Y (2005) Vertical stratification of leaf-beetle assemblages (Coleoptera: Chrysomelidae) in two forest types in Panama. Journal of Tropical Ecology 21: 329–336. https://doi.org/10.1017/S0266467405002300

[B22] D’Alessandro P Grobbelaar E Biondi M (2012) Revision of the genus *Stegnaspea* Baly with descriptions of five new species from southern Africa (Coleoptera, Chrysomelidae, Galerucinae, Alticini). Insect Systematics & Evolution 43: 11–33. https://doi.org/10.1163/187631212X626032

[B23] D’Alessandro P Urbani F Biondi M (2014) Biodiversity and biogeography in Madagascar: revision of the endemic flea beetle genus *Neodera* Duvivier, 1891 with description of 19 new species (Coleoptera, Chrysomelidae, Galerucinae, Alticini). Systematic Entomology 39: 710–748. https://doi.org/10.1111/syen.12082

[B24] D’Alessandro P Samuelson A Biondi M (2016) Taxonomic revision of the genus *Arsipoda* Erichson, 1842 (Coleoptera, Chrysomelidae) in New Caledonia. European Journal of Taxonomy 230: 1–61. https://doi.org/10.5852/ejt.2016.230

[B25] D’Alessandro P Frasca R Grobbelaar E Iannella M Biondi M (2017) Systematics and biogeography of the Afrotropical flea beetle subgenus Blepharidina (Afroblepharida) Biondi and D’Alessandro, with description of seven new species (Coleoptera, Chrysomelidae, Galerucinae, Alticini). Insect Systematics & Evolution 2017. https://doi.org/10.1163/1876312X-00002182

[B26] Damaška A Konstantinov A (2016) A new species of *Cangshanaltica* Konstantinov et al., a mossinhabiting flea beetle from Thailand (Coleoptera: Chrysomelidae: Galerucinae: Alticini). Zootaxa 4107(1): 093–097. https://doi.org/10.11646/zootaxa.4107.1.7 10.11646/zootaxa.4107.1.7 27394809

[B27] Davis AJ Sutton SL Brendell MJD (2011) Vertical distribution of beetles in a tropical rainforest in Sulawesi: the role of the canopy in contributing to biodiversity. Sepilok Bulletin 13 and 14: 59–83.

[B28] Döberl M (1986) Die spermathek als bestimmungshilfe bei den Alticinen. Entomologische Blätter 82: 3–14.

[B29] Döberl M (2010) Beitrag zur Kenntnis der afrotropischen Arten von Altica Geoffroy, 1762 unter Ausschluss der Arten Madagaskars (Coleoptera: Chrysomelidae: Alticinae). Entomologische Zeitschrift 120: 51–72.

[B30] Döberl M (2012) Alticinae (Coleoptera: Chrysomelidae) of Socotra Island. In: Hájek J Beždek J (Eds) Insect biodiversity of the Socotra Archipelago. Acta Entomologica Musei Nationalis Pragae 52 (Supplementum 2), 429–447.

[B31] Evenhuis NL (2016) The insect and spider collections of the world website http://hbs.bishopmuseum.org/codens

[B32] Furth DG (1982) The Metafemoral Spring of Flea Beetles (Chrysomelidae: Alticinae) Spixiana 7: 11–27.

[B33] Furth DG Suzuki K (1994) Character correlation studies of problematic genera of Alticinae in relation to Galerucinae (Coleoptera: Chrysomelidae). In: Furth DG (Ed.) Proceedings of the Third International Symposium on the Chrysomelidae, Beijing (China), 1992. Backhuys Publishers, Leiden, 116–135.

[B34] Furth DG Suzuki K (1998) Studies of Oriental and Australian Alticinae genera based on the comparative morphology of the metafemoral spring, genitalia, and hind wing venation. In: Biondi M Daccordi M Furth DG (Eds) Proceedings of the Fourth International Symposium on the Chrysomelidae. XX International Congress of Entomology, Firenze (Italy), 1996. Museo Regionale di Scienze Naturali, Torino, 91–124.

[B35] Furth DG Longino JT Paniagua M (2003) Survey and quantitative assessment of flea beetle diversity in a Costa Rican rainforest (Coleoptera: Chrysomelidae: Alticinae) In: Furth DG (Ed.) Special Topics in Leaf Beetle Biology. Proceedings of the Fifth International Symposium on the Chrysomelidae. XXI International Congress of Entomology, Iguassu Falls (Brazil), August 2000. Pensoft Publisher, Sofia-Moscow, 1–23.

[B36] Insektoid.Info (2017) http://insektoid.info/insecta/coleoptera/chrysomelidae/alticini/

[B37] Jolivet P Verma KK (2002) Biology of leaf beetles. Intercept, Andover, 332 pp.

[B38] Konstantinov A Chamorro ML Prathapan KD Ge S-Q Yang X-K (2013) Moss-inhabiting flea beetles (Coleoptera: Chrysomelidae: Galerucinae: Alticini) with description of a new genus from Cangshan, China. Journal of Natural History 47(37–38): 2459–2477. https://doi.org/10.1080/00222933.2012.763068

[B39] Nadein KS (2012) Catalogue of Alticini genera of the World (Coleoptera: Chrysomelidae). Beetles and Coleopterists website, Zoological Institute, Saint-Petersburg. http://www.zin.ru/Animalia/Coleoptera/eng/alticinw.htm

[B40] Nadein KS Betz O (2016) Jumping mechanisms and performance in beetles. I. Flea beetles (Coleoptera: Chrysomelidae: Alticini). Journal of Experimental Biology 219: 2015–2027. https://doi.org/10.1242/jeb.140533 27385755 10.1242/jeb.140533

[B41] Nadein KS Beždek J (2014) Galerucinae Latreille 1802. In: Leschen RAB Beutel RG (Eds) Handbook of Zoology, Volume 4/40: Coleoptera, Beetles, Volume 3: Morphology and Systematics (Phytophaga). Walter de Gruyter Publishers, Berlin, 251–259.

[B42] Ruan Y Konstantinov AS Prathapan KD Yang X (2017) Contributions to the knowledge of Chinese flea beetle fauna (II): *Baoshanaltica* new genus and *Sinosphaera* new genus (Coleoptera, Chrysomelidae, Galerucinae, Alticini). ZooKeys 720: 103–120. https://doi.org/10.3897/zookeys.720.12715 10.3897/zookeys.720.12715 PMC5740446 29290728

[B43] Suzuki K (1988) Comparative morphology of the internal reproductive system of the Chrysomelidae (Coleoptera). In: Jolivet P Petitpierre E Hsiao TH (Eds) Biology of Chrysomelidae. Series Entomologica 42. Kluwer Academic, Dordrecht, 317–355. https://doi.org/10.1007/978-94-009-3105-3_19

[B44] Wagner T (1999) Arboreal chrysomelid community structure and faunal overlap between different types of forests in Central Africa. In: Cox ML (Ed.) Advances in Chrysomelidae Biology 1. Backhuys Publishers, Leiden, 247–270.

[B45] Wagner T (2000) Influence of Forest Type and Tree Species on Canopy-Dwelling Beetles in Budongo Forest, Uganda. Biotropica 32(3): 502–514. https://doi.org/10.1111/j.1744-7429.2000.tb00496.x

[B46] Wagner T (2001) Seasonal changes in the canopy arthropod fauna in *Rinorea beniensis* in Budongo Forest, Uganda. Plant Ecology 153: 169–178. https://doi.org/10.1023/A:1017514417913

